# Influence of Sire Breed on the Interplay among Rumen Microbial Populations Inhabiting the Rumen Liquid of the Progeny in Beef Cattle

**DOI:** 10.1371/journal.pone.0058461

**Published:** 2013-03-08

**Authors:** Emma Hernandez-Sanabria, Laksiri A. Goonewardene, Zhiquan Wang, Mi Zhou, Stephen S. Moore, Le Luo Guan

**Affiliations:** 1 Department of Agricultural, Food and Nutritional Science, University of Alberta, Edmonton, Alberta, Canada; 2 The University of Queensland Centre for Animal Science, Queensland Alliance for Agriculture and Food Innovation, St. Lucia, Queensland, Australia; National University of Singapore, Singapore

## Abstract

This study aimed to evaluate whether the host genetic background impact the ruminal microbial communities of the progeny of sires from three different breeds under different diets. Eighty five bacterial and twenty eight methanogen phylotypes from 49 individuals of diverging sire breed (Angus, ANG; Charolais, CHA; and Hybrid, HYB), fed high energy density (HE) and low energy density (LE) diets were determined and correlated with breed, rumen fermentation and phenotypic variables, using multivariate statistical approaches. When bacterial phylotypes were compared between diets, ANG offspring showed the lowest number of diet-associated phylotypes, whereas CHA and HYB progenies had seventeen and twenty-three diet-associated phylotypes, respectively. For the methanogen phylotypes, there were no sire breed-associated phylotypes; however, seven phylotypes were significantly different among breeds on either diet (*P*<0.05). Sire breed did not influence the metabolic variables measured when high energy diet was fed. A correlation matrix of all pairwise comparisons among frequencies of bacterial and methanogen phylotypes uncovered their relationships with sire breed. A cluster containing methanogen phylotypes M16 (*Methanobrevibacter gottschalkii*) and M20 (*Methanobrevibacter smithii*), and bacterial phylotype B62 (*Robinsoniella* sp.) in Angus offspring fed low energy diet reflected the metabolic interactions among microbial consortia. The clustering of the phylotype frequencies from the three breeds indicated that phylotypes detected in CHA and HYB progenies are more similar among them, compared to ANG animals. Our results revealed that the frequency of particular microbial phylotypes in the progeny of cattle may be influenced by the sire breed when different diets are fed and ultimately further impact host metabolic functions, such as feed efficiency.

## Introduction

The rumen ecosystem has been found to be a complex system of vital importance for the productivity of ruminant livestock. Although the composition of rumen microbiota across individuals has been demonstrated to include a stable core [1], [2], the animal-to-animal differences in the abundance of particular genera [3], [4] indicate that rumen microbiota can be influenced by a number of environmental factors [5], [6], [7]. The development of molecular tools have revealed the extraordinary richness of bacterial species in the rumen [8] and metagenomic analysis have provided additional knowledge of the bacterial community and their potential functions impacting host performance [9], [10]. While activities and interactions among bacterial communities in the rumen appear to be formerly examined [11], little is known about the fluctuations in microbial populations influenced by the host genotype. Previous studies have demonstrated that the composition of the human gut bacterial community is host-specific [12], [13], and that the presence of particular microbial groups in the gastrointestinal tract may be determined by the host influence [14]. Thus, the host effects on the gut microbial ecosystem cannot be neglected [15]. The effect of host genetics on the gut microbiota has been reported in studies conducted in related individuals [16]. Research in humans revealed associations between similarity of bacterial profiles and genetic relatedness of the subjects [17], [18]. Further studies in mice showed high similarity of the gut microbiota composition within mouse lines [19]. Moreover, variations at a given host locus have been associated with variations in the abundance of particular microbial taxa [20]. Hence, host genetics can have an effect on the composition of its associated gut microbiota [21]. Nevertheless, the particular host mechanisms responsible for the variations in the microbial populations and their interactions in the rumen have not been explored and defined.

In the present study, we hypothesised that sire breed may impact ruminal microbial groups and bacterial-methanogen interactions of the progeny. The relevance of revealing the relationships with sire breed is enlarged, because selection strategies are mainly geared towards improving the efficiency of the breeding sires, as most of the genetic improvement is achieved when sires pass on their characteristics to their offspring. Thus, sire breed was considered relevant for the identification of potential relationships among microbial groups, which could be used either as a marker for productivity or potentially inherited to the offspring. Because the effect of genotype can be assessed more accurately under specific environmental conditions [19], [22], we screened the diversity of the bacterial and methanogen populations in the rumen liquid from the offspring of sires from three different breeds: Angus (ANG), Charolais (CHA) and Hybrid (HYB); all individuals were fed two different diets (low energy diet, LE and high energy diet, HE) and were under equal management conditions. Diverse multivariate statistical approaches [10], [23], [24] permitted establishing the frequency of diet–associated and sire breed–associated microbial phylotypes, from both bacteria and methanogens. Bacterial phylotypes were selected for validation and independently analysed for each breed, due to their unique functions, as suggested by previous studies [2], [10]. Further, we explored the potential relationships among phenotypic characteristics of the host (feed efficiency) with functional microbiota (breed- and diet-associated phylotypes) and genotypic background of the host (sire breed).

## Results

### Evaluation of Sire Breed Effect on Bacterial and Methanogen Phylotypes under Diverging Diets

**Figure 1:**
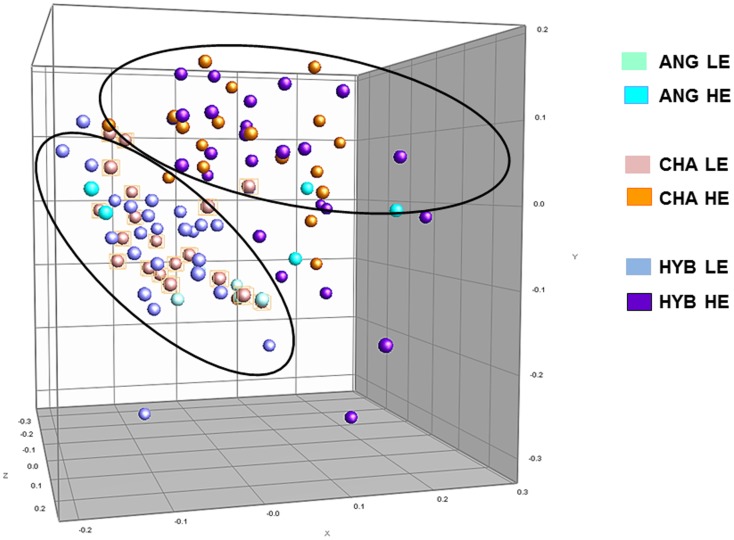
Multidimensional Scaling Analysis of the bacterial profiles generated from the rumen fluid of 49 steers, progeny of ANG, CHA or HYB sires. DNA from forty-nine steers fed low energy (LE) density diet and then switched to high energy (HE) density diet, was amplified using primers HDA1-GC and HDA2 (22 to 55% DGGE). Colours represent a particular sire breed: light blue, ANG fed LE diet; cyan blue, ANG fed HE diet; light pink, CHA fed LE diet; orange, CHA fed HE diet; azure blue, HYB fed LE diet, and purple, HYB fed HE diet. The comparison of the PCR-DGGE profiles was generated with the Bionumerics software package using UPGMA (unweighted pair-group) method as described in the text; comparison was optimised upon calculation of the best values for tolerance.

Diversity of the bacterial and methanogen communities inhabiting the rumen fluid of steers fed LE and HE diet was screened, and determined from previous studies (data not shown) [10], [25]. Methanogen phylotypes tended to group by breed under HE diet (data not shown); while bacterial phylotypes tended to cluster by diet based on multidimensional scaling (MDS) analysis, (Figure 1).

Twenty-four bacterial phylotypes were significantly different among sire breeds when LE diet was fed. Four phylotypes were ANG-associated (present in all steers of this breed), while specific associations with the other two sire breeds were not observed. Under HE diet, thirty-seven phylotypes were significantly different; six of them were ANG-associated, and one was CHA-associated. No phylotypes were specifically associated with HYB individuals (Table 1). However, when phylotypes were compared between diets, ANG steers showed the lowest number of diet-associated phylotypes (one), whereas CHA and HYB had seventeen and twenty-three, respectively (Table 2).

**Table 1:** Taxonomic identification of breed-associated bacterial phylotypes in rumen liquid of steers feed diverging diets (n = 49).

Phylotype	LE DIET				HE DIET				Closest related taxon (GenBank Accession no.)
	Breed			*P* value	Breed			*P* value	
	Frequency in ANG (%, n = 5)	Frequency in CHA (%, n = 19)	Frequency in HYB (%, n = 25)		Frequency in ANG (%, n = 5)	Frequency in CHA (%, n = 19)	Frequency in HYB (%, n = 25)		
1	20.0	21.1	8.0	0.07	60.0	31.6	20.0	0.02	*Prevotella* sp. (AF218619)
2	20.0	36.8	16.0	0.03	100.0	57.9	24.0	0.0003	*Prevotella* sp. (AF218619)
3	20.0	57.9	56.0	0.03	80.0	63.2	68.0	0.08	*Prevotella maculosa* strain W1609 (EF534315)
4	100.0	42.1	68.0	0.004	40.0	42.1	40.0	0.09	-*
5	60.0	78.9	52.0	0.02	80.0	78.9	56.0	0.03	Uncultured *Succinivibrio* sp. clone EMP_B23 (EU794184)
6	80.0	68.4	88.0	0.04	80.0	63.2	80.0	0.05	*Prevotella* sp. BP1-56 (AB501155)
9	20.0	26.3	24.0	0.11	40.0	0.0	12.0	0.01	*Blautia* sp. BM-C2-0 (GQ456220)
10	20.0	21.1	28.0	0.10	40.0	5.3	16.0	0.03	*Clostridium symbiosum* strain 69 (EF025909)
11	20.0	15.8	12.0	0.13	60.0	15.8	16.0	0.01	*Prevotella oulorum* strain WPH 179 (NR_029147)
12	40.0	31.6	40.0	0.08	40.0	0.0	12.0	0.01	*Prevotella denticola* clone WWP_SS6_P23 (GU409439)
25	20.0	15.8	8.0	0.10	40.0	10.5	16.0	0.05	*Prevotella oulora* (L16472.2)
30	0.0	26.3	20.0	0.08	40.0	10.5	12.0	0.05	*Prevotella maculosa* strain GEJ21 (GU561342)
34	0.0	47.4	24.0	0.01	40.0	26.3	8.0	0.02	*Ruminococcus gauvreaui* strain CCRI 16110
36	0.0	31.6	32.0	0.04	40.0	10.5	28.0	0.03	*Butyrivibrio fibrisolvens* strain H15 (EU887842)
37	40.0	31.6	28.0	0.08	60.0	26.3	32.0	0.04	Uncultured *Prevotella* sp. clone Sew1-325 (FJ219872)
38	40.0	31.6	64.0	0.01	60.0	52.6	52.0	0.08	*Clostridium indolis* (AF028351)
39	40.0	63.2	44.0	0.04	40.0	42.1	28.0	0.06	Prevotellaceae
40	60.0	73.7	56.0	0.04	40.0	42.1	52.0	0.07	*Vibrio* sp. WH134 (FJ847833)
41	100.0	36.8	32.0	0.002	60.0	36.8	40.0	0.06	*Ruminococcus* sp. ZS2-15 (FJ889653)
42	60.0	57.9	72.0	0.06	100.0	63.2	80.0	0.03	*Prevotella* sp. 152R-1a (DQ278861)
44	60.0	52.6	56.0	0.08	100.0	52.6	60.0	0.02	Uncultured *Prevotella* sp. clone JD9 (FJ268952)
46	80.0	73.7	60.0	0.06	100.0	68.4	64.0	0.04	Uncultured *Prevotella* sp. clone JD9 (FJ268952)
47	100.0	63.2	84.0	0.02	80.0	63.2	72.0	0.08	*Hespellia porcina* strain PC80 (NR_025206)
49	80.0	78.9	76.0	0.12	40.0	57.9	76.0	0.02	Uncultured *Prevotella* sp. clone Gull85-50 (FJ220908)
50	80.0	73.7	80.0	0.11	60.0	100.0	80.0	0.006	*Prevotella ruminicola* strain TC2-3 (AF218617)
51	80.0	73.7	96.0	0.02	60.0	84.2	72.0	0.05	*Robinsoniella peoriensis* strain HGUE-09/9434 (GU322806)
55	100.0	84.2	80.0	0.11	100.0	63.2	72.0	0.04	Uncultured *Succinivibrio* sp. clone EMP_J46 (EU794280)
57	80.0	89.5	76.0	0.07	100.0	84.2	68.0	0.04	*Eubacterium xylanophilum* (L34628)
58	60.0	63.2	72.0	0.07	80.0	89.5	64.0	0.02	*Moryella indoligenes* strain AIP 220.04 (DQ377947)
59	100.0	47.4	68.0	0.009	100.0	84.2	76.0	0.08	Uncultured *Succinivibrio* sp. clone EMP_V30 (EU794288)
60	60.0	94.7	92.0	0.03	100.0	89.5	80.0	0.11	*Anaerophaga thermohalophila* strain Fru22 (NR_028963)
64	60.0	42.1	40.0	0.06	0.0	78.9	72.0	0.0006	*Butyrivibrio fibrisolvens* strain H15 (EU887842)
66	40.0	68.4	84.0	0.01	80.0	94.7	68.0	0.01	Uncultured *Succinivibrio* sp. clone EMP_B23 (EU794184)
67	80.0	68.4	72.0	0.10	40.0	68.4	68.0	0.05	*Succinivibrio dextrinosolvens* strain 0554 (NR_026476)
69	40.0	73.7	64.0	0.04	60.0	78.9	44.0	0.01	Uncultured *Prevotella* sp. clone 3083 (FJ976203)
70	40.0	73.7	64.0	0.04	60.0	63.2	56.0	0.08	*Succinivibrio dextrinosolvens* strain 0554 (NR_026476)
71	20.0	52.6	76.0	0.004	80.0	57.9	40.0	0.02	*Coprococcus eutactus* strain ATCC 27759 (EF031543)
72	0.0	57.9	76.0	0.0007	60.0	26.3	48.0	0.02	*Clostridium indolis* (AF028351)
73	60.0	57.9	52.0	0.08	60.0	36.8	28.0	0.04	Uncultured *Succinivibrio* sp. clone EMP_V30 (EU794288)
74	20.0	42.1	28.0	0.05	40.0	21.1	40.0	0.04	Uncultured *Succinivibrio* sp. clone EMP_V30 (EU794288)
76	0.0	31.6	16.0	0.04	0.0	57.9	60.0	0.004	*Moryella indoligenes* strain AIP 220.04 (DQ377947)
80	0.0	5.3	8.0	0.31	0.0	42.1	48.0	0.01	*Ruminococcus bromii* strain YE282 (DQ882649)
83	0.0	0.0	20.0	0.03	60.0	26.3	44.0	0.03	*Selenomonas ruminantium*, strain: S211 (AB198441.1)
84	20.0	10.5	20.0	0.10	40.0	5.3	8.0	0.03	*Bifidobacterium ruminantium* strain KCTC 3425 (GU361831)

* Not determined.

**Table 2:** Taxonomical identification of diet-associated bacterial phylotypes within particular breed cohorts (n = 49).

	ANG (n = 5)			CHA (n = 19)			HYB (n = 25)			
Phylotype	Frequency in HE (%)	Frequency inLE (%)	P value	Frequency in HE (%)	Frequency inLE (%)	P value	Frequency in HE (%)	Frequency inLE (%)	P value	Closest related taxon (GenBank Accession no.)
2	100.0	20.0	0.02							*Prevotella* sp. (AF218619)
4							40.0	68.0	0.03	-*
7							36.0	72.0	0.009	*Lactobacillus sp.* DI71 (AB290831)
9				0.0	26.3	0.02				*Blautia* sp. BM-C2-0 (GQ456220)
12				0.0	31.6	0.01	12.0	40.0	0.02	*Prevotella denticola* clone WWP_SS6_P23 (GU409439)
14							4.0	28.0	0.02	*Lachnospiraceae genomosp*. C1 (AY278618)
32							4.0	28.0	0.02	*Prevotella ruminicola* (AB219152)
35							12.0	36.0	0.04	Uncultured *Prevotella* sp. clone JD9 (FJ268952)
40				42.1	73.7	0.04				*Vibrio* sp. WH134 (FJ847833)
48				89.5	52.6	0.01				*Lactobacillus* sp. DI71 (AB290831)
50				100.0	73.7	0.02				*Prevotella ruminicola* strain TC2-3 (AF218617)
51							72.0	96.0	0.02	*Robinsoniella peoriensis* strain HGUE-09/9434 (GU322806)
54							92.0	56.0	0.004	*Succinivibrio dextrinosolvens* strain 0554 (NR_026476)
58				89.5	63.2	0.05				*Moryella indoligenes* strain AIP 220.04 (DQ377947)
59				84.2	47.4	0.02				Uncultured *Succinivibrio* sp. clone EMP_V30 (EU794288)
63							76.0	96.0	0.04	*Eubacterium rectale* ATCC 33656 (CP001107)
64				78.9	42.1	0.02	72.0	40.0	0.02	*Butyrivibrio fibrisolvens* strain H15 (EU887842)
65				73.7	26.3	0.004	68.0	28.0	0.004	*Robinsoniella peoriensis* strain HGUE-09/9434 (GU322806)
66				94.7	68.4	0.04				Uncultured *Succinivibrio* sp. clone EMP_B23 (EU794184)
71							40.0	76.0	0.009	*Coprococcus eutactus* strain ATCC 27759 (EF031543)
72				26.3	57.9	0.04	48.0	76.0	0.03	*Clostridium indolis* (AF028351)
73							28.0	52.0	0.05	Uncultured *Succinivibrio* sp. clone EMP_V30 (EU794288)
75							56.0	20.0	0.008	–
76							60.0	16.0	0.001	*Moryella indoligenes* strain AIP 220.04 (DQ377947)
77				63.2	21.1	0.009	56.0	20.0	0.008	*Succinivibrio dextrinosolvens* strain 0554 (NR_026476)
78				63.2	21.1	0.009	52.0	12.0	0.002	–
80				42.1	5.3	0.009	48.0	8.0	0.002	*Ruminococcus bromii* strain YE282 (DQ882649)
81							32.0	4.0	0.01	–
82				89.5	0.0	<0.0001	52.0	4.0	<0.0001	–
83				26.3	0.0	0.02	44.0	20.0	0.05	*Selenomonas ruminantium*, strain: S211 (AB198441.1)
85				52.6	5.3	0.002	52.0	12.0	0.002	–

For the methanogen phylotypes, there were no sire breed-associated specific phylotypes; however, seven phylotypes were significantly different among breeds on each diet (*P*<0.05) (Table 3). Different phylotypes belonging to *Methanobrevibacter* sp. were associated to changes in diet on the three breeds (Tables 3 and 4). In ANG offspring, three phylotypes were impacted by diet change and *Methanobrevibacter olleyae* was LE-associated. In CHA progeny, the frequency of nine methanogen phylotypes was significantly different between both diets; *Methanobrevibacter* sp. AbM4 and *Methanobrevibacter smithii* were CHA-associated under HE diet (present in the entire CHA cohort). In HYB steers, twelve phylotypes were significantly different between diets (*P*<0.05) and five of them were exclusive of HE and one of LE, respectively (Table 4). The frequencies of *Methanobrevibacter smithii* SM9 and *Methanobrevibacter* sp. AbM4 in HYB offspring were high when HE diet was fed.

**Table 3:** Taxonomic identification of breed-associated methanogen phylotypes in rumen liquid of steers feed diverging diets (n = 49).

Phylotype	LE DIET				HE DIET				Closest related taxon (GenBank Accession no.)	
	Breed			*P* value	Breed			*P* value		
	Frequency in ANG(%, n = 5)	Frequency inCHA(%, n = 19)	Frequency in HYB (%, n = 25)		Frequency in ANG(%, n = 5)	Frequency in CHA(%, n = 19)	Frequency in HYB (%, n = 25)			
5	20.0	15.8	0.0	0.02	0.0	0.0	0.0	1.00	*Methanobrevibacter gottschalkii* strain HO	
6	20.0	31.6	24.0	0.09	60.0	5.3	12.5	0.005	*Methanosphaera stadtmanae* (AY196684)	
7	40.	42.1	68.0	0.02	60.0	57.9	60.0	0.09	*Methanosphaera stadtmanae* (AY196684)	
8	60.0	57.9	44.0	0.05	60.0	68.4	76.0	0.07	*Methanobrevibacter gottschalkii* strain HO	
9	60.0	36.8	28.0	0.04	0.0	0.0	4.0	0.51	Methanogenic archaeon SRmetG36	
11	60.0	21.1	20.0	0.02	100.0	89.5	88.0	0.21	*Methanobrevibacter* sp. AbM4 (AJ550156)	
16	100.0	78.9	92.0	0.08	60.0	73.7	88.0	0.03	*Methanobrevibacter gottschalkii* strain HO	
19	0.0	0.0	16.	0.06	60.0	10.5	28.0	0.009	*Methanobrevibacter* sp. AbM4 (AJ550156)	
20	100.0	94.7	88.0	0.21	60.0	89.5	84.0	0.05	*Methanobrevibacter smithii* (AY196669)	
21	100.0	84.2	92.0	0.15	20.0	63.2	56.0	0.02	*Methanobrevibacter olleyae* (AY615201)	
27	0.0	0.0	0.0	1.00	40.0	78.9	72.0	0.03	*Methanobrevibacter smithii* SM9 (AJ009958)	

**Table 4:** Taxonomical identification of diet-associated methanogen phylotypes within particular breed cohorts (n = 49).

	ANG (n = 5)			CHA (n = 19)			HYB (n = 25)			Closest related taxon (GenBank Accession no.)
Phylotype	Frequency in HE (%)	Frequency in LE (%)	P value	Frequency in HE (%)	Frequency in LE (%)	P value	Frequency in HE (%)	Frequency in LE (%)	P value	
1	0.0	80.0	0.02	0.0	47.4	0.0006	0.0	72.0	<0.0001	–
3				89.5	31.6	0.0003	84.0	48.0	0.01	*Methanobrevibacter thaueri* strain CW (U55236)
6				5.3	31.6	0.04				*Methanosphaera stadtmanae* (AY196684)
7										*Methanosphaera stadtmanae* (AY196684)
8							76.0	44.0	0.02	*Methanobrevibacter gottschalkii* strain HO
9				0.0	36.8	0.004	4.0	28.0	0.02	Methanogenic archaeon SRmetG36
11				89.5	21.1	<0.0001	88.0	20.0	<0.0001	*Methanobrevibacter* sp. AbM4 (AJ550156)
12							24.0	0.0	0.01	–
18				21.1	0.0	0.05	16.0	0.0	0.05	*Methanobrevibacter smithii* PS (U55233)
21	20.0	100.0	0.02				56.0	92.0	0.004	*Methanobrevibacter olleyae* (AY615201)
22				47.4	94.7	0.002	60.0	96.0	0.002	*Methanobrevibacter smithii* ATCC 35061 (CP000678)
24	80.0	0.0	0.02	84.2	0.0	<0.0001	80.0	0.0	<0.0001	*Methanobrevibacter* sp. AbM4 (AJ550156)
25							16.0	0.0	0.05	–
27				78.9	0.0	<0.0001	72.0	0.0	<0.0001	*Methanobrevibacter smithii* SM9 (AJ009958)

### Characterisation of the Relationships among Metabolic Indicators of the Microbial Interactions and Sire Breed

To obtain a comprehensive evaluation of the impact of sire breed on the microbial activities and their relationship with host characteristics, proportions of ruminal volatile fatty acid (VFA) and concentrations of ruminal ammonia-nitrogen (NH_3_-N) under differing diet conditions were assessed. Hence, secondary information on whether sire breed influenced the microbial activities in the rumen was provided. In addition, phenotypic traits such as average daily gain (ADG), dry matter intake (DMI), feed conversion ratio (FCR) and residual feed intake (RFI) were variables selected for studying the physiological mechanisms underlying feed utilisation among breeds, and ultimately reflecting physiological differences in basic metabolic processes of the energetic metabolism of the host [26], [27]. Ammonia-N concentration was higher in ANG offspring (*P*<0.05) when LE diet was fed. Trends for increased isovalerate proportion and low feed conversion ratio (FCR, *P*<0.10) were also recorded for this diet. Sire breed did not influence all the additional variables measured in LE diet (Table 5). Similarly, no significant differences in the phenotypic/rumen fermentation measurements were detected among breeds when HE diet was fed (data not shown). Thus, correlation analysis was also performed within sire breed, for the variables mentioned above. Under LE diet, no significant associations were detected among phenotypic and fermentation variables on any of the three breeds (data not shown).

**Table 5:** Phenotypic indicators of metabolic differences (RFI, DMI and FCR) and ruminal metabolic measurements in steers differing breed (n = 49) in LE.

Variable	Breed			*P* value		
	Angus	Charolais	Hybrid			
	Mean ± SEM	Mean ± SEM	Mean ± SEM			
	(n = 5)	(n = 19)	(n = 25)			
**Acetate** **(%)** *	57.30±2.23	54.81±1.15	53.96±0.99	**0.39**		
**Propionate** **(%)** *	28.10±2.59	32.89±1.33	33.32±1.16	**0.18**		
**Isobutyrate** **(%)** *	0.98±0.11	0.82±0.06	0.91±0.05	**0.30**		
**Isovalerate** **(%)** *	2.99±0.39	2.02±0.20	2.09±0.17	**0.09**		
**Acetate : Propionate ratio**	2.18±0.26	1.71±0.13	1.73±0.12	**0.27**		
**Ammonia (mM)**	0.16±0.002	0.09±0.01	0.11±0.01	**0.01**		
**Dry Matter Intake (DMI) (kg DM)**	7.38±0.43	7.51±0.22	7.97±0.19	**0.18**		
**Feed Conversion Ratio (FCR)**	5.76±0.39	6.22±0.20	6.68±0.17	**0.06**		
**Residual Feed Intake (RFI)**	−0.28±0.31	−0.06±0.16	0.24±0.14	**0.19**		

* Values are given as a proportion of the total VFA concentration.

### Analysis of the Influence of Sire Breed on the Interplay among Ruminal Microbial Populations under Different Diets

As sire breed-associated differences among specific bacterial and methanogen phylotypes were observed, total bacteria, total methanogens, and three selected bacterial phylotypes were validated and associated to each sire breed under different diets (Tables 6 and 7). Bacterial phylotypes quantified were: *Robinsoniella peoriensis-*like sp. (associated with the three breeds in LE), *Eubacterium* sp. (associated with ANG steers in both diets) and *Succinivibrio dextrinosolven-*like sp. (associated with HYB steers in HE). Under LE diet, high population of total methanogens tended to correlate with low FCR in ANG offspring (*P*<0.10), whereas high proportion of *Robinsoniella* sp. was associated with high ruminal ammonia (*P*<0.05, Supplementary Table S1). In HYB offspring, a trend for high total methanogens was associated with high RFI (low efficiency, *P*<0.10). No particular associations were detected between bacterial phylotypes and phenotypic measurements in CHA steers when LE was supplied. When the diet was switched to HE, high population of methanogens was correlated with low RFI (high efficiency, *P*<0.05) in ANG steers, and the proportion of *Robinsoniella* sp. tended to be positively correlated with the same trait (*P*<0.10, Supplementary Table S2) in HYB progeny.

**Table 6:** Particular bacterial species and methanogen population in steers differing breed (n = 49) in LE.

Variable	Breed			*P* value
	Angus	Charolais	Hybrid	
	Mean ± SEM	Mean ± SEM	Mean ± SEM	
	(n = 5)	(n = 19)	(n = 25)	
**Total bacteria**	7.38E10±2.31E10	3.08E10±1.186E10	5.72E10±1.03E10	**0.14**
***Succinivibrio*** ** sp. (%)**	7.32±9.12	29.57±4.68	17.28±4.08	**0.05**
***Eubacterium*** ** sp. (%)**	0.03±0.11	0.08±0.05	0.19±0.05	**0.24**
***Robinsoniella*** ** sp. (%)**	0.002±0.002	0.003±9.55E−4	0.004±8.32E−4	**0.47**
**Total Methanogens**	3.82E7±6.59E6	1.79E7±3.38E6	2.43E7±2.94E6	**0.03**

**Table 7:** Particular bacterial species and methanogen population in steers differing breed (n = 49) in HE.

Variable	Breed			*P* value
	Angus	Charolais	Hybrid	
	Mean ± SEM	Mean ± SEM	Mean ± SEM	
	(n = 5)	(n = 19)	(n = 25)	
**Total bacteria**	8.53E10±4.47E11	1.34E11±2.29E11	4.20E11±2.00E11	**0.59**
***Succinivibrio*** ** sp. (%)**	0.07±1.13	1.23±0.58	0.08±0.51	**0.31**
***Eubacterium*** ** sp. (%)**	0.41±0.12	0.08±0.06	0.12±0.05	**0.05**
***Robinsoniella*** ** sp. (%)**	0.005±0.008	0.01±0.004	0.002±0.004	**0.32**
**Total Methanogens**	2.87E7±1.15E7	2.07E7±5.91E7	1.28E7±5.15E7	**0.36**

**Figure 2:**
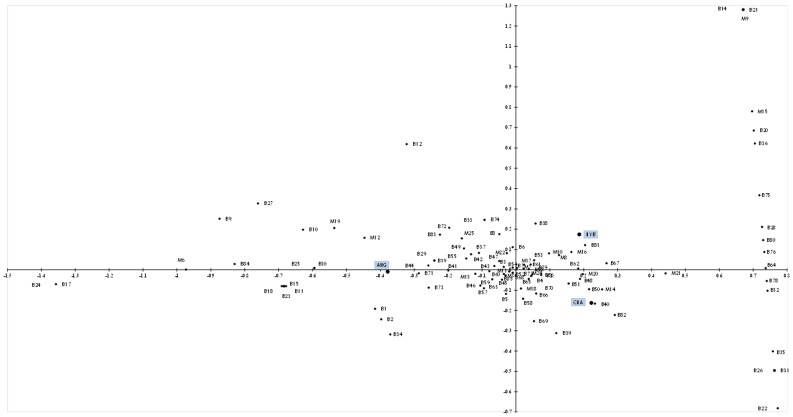
Correspondence Analysis (CA) plot displaying the interactions among frequencies of bacterial and methanogen phylotypes and sire breed. Structural relationships among frequencies were displayed in a data cloud, where Dimension 1 indicated the frequency of either methanogen or bacterial phylotypes (column coordinates) while Dimension 2 depicts the associations of these observed frequencies and breed (row coordinates).

Further multivariate statistical approaches were employed to obtain evidence of the role of sire breed on the relationships between ruminal bacteria and methanogens. Principal Component Analysis (PCA) failed to show how frequency of specific bacterial phylotypes varied in parallel with shifts on the methanogen phylotypes, under both diets (data not shown). Therefore Correspondence Analysis (CA) [23] was used to reveal whether the frequencies of the detected bacterial and methanogen phylotypes tended to overlap with diet variations. Correspondence analysis is most effective when the data matrix is large, so that visual inspection or simple statistical analysis cannot reveal its structure. In the graphical display of the frequencies, each row and each column of the contingency tables obtained with PROC FREQ was depicted as a point. Hence, for each sire breed there was a cloud of profile points representing phylotypes, which values add up to 1. These points have a centroid (i.e., the average value of all the points) and a distance (Chi-square distance) between profile points. Each profile point contributes to the deviation from the averaged distance of the whole cloud of the data points from a particular breed (namely “inertia”). The CA reduces the dimension of the data by identifying the deviations of the data points from the expected value; thus, the total variance is decomposed in a lower dimensional representation of the variables. This method allowed determining some potential associations between phylotypes, based on the sire breed (Figure 2). ANG makes a relatively small contribution to the chi-square statistic and does not contribute to the inertia in Dimension 2. This is to say, all the data points from the frequencies of the phylotypes present in ANG in Dimension 2 have a small value. The horizontal dimension (Dim1) seemed to be determined by the ANG vs CHA steers in HE (Figure 2), whereas in the vertical dimension (Dim2) was defined by HYB and CHA progenies.

**Figure 3:**
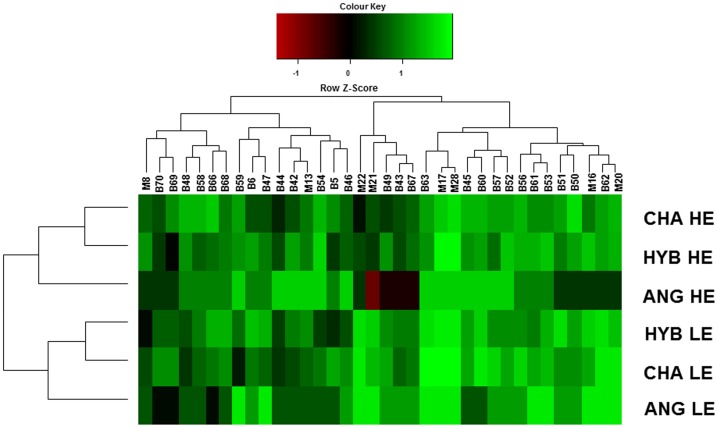
Heat map of correlations between frequencies of bacterial and methanogen phylotypes and their relationship with diet (LE/HE) and sire breed (ANG, CHA, HYB). Each square represents the Spearman’s correlation coefficient between the frequencies of the phylotype in the column with the frequency of the phylotype in the row. Order of phyltypes is determined as in a hierarchical cluster analysis. Self-correlations are identified in dark colour.

A correlation matrix of all pairwise comparisons [24] among frequencies of bacterial and methanogen phylotypes uncovered additional relationships associated with sire breed (Figure 3). For instance, methanogen phylotypes M16 (*Methanobrevibacter gottschalkii* and M20 (*Methanobrevibacter smithii*)), and bacterial phylotype B62 (*Robinsoniella* sp.) were clustered together in ANG offspring when LE diet was fed, as they all had frequencies above the average. Instead, when HE was provided, bacterial phylotypes B42 (*Prevotella* sp.) and M13 (*Methanobrevibacter gottschalkii*) were allocated within a common cluster for this sire breed. Progeny of ANG and CHA sires showed similarities among the frequencies of phylotypes M16, B62 and M20, under LE diet. Phylotypes M17 (*Methanobrevibacter ruminantium*) and M28 (*Methanobrevibacter gottschalkii*) had similar frequencies in HYB and CHA offspring under HE diet. The clustering of the frequencies of all the phylotypes from the three breeds revealed that the frequencies of all the detected phylotypes in CHA and HYB progenies were more similar between these two breeds, in comparison with individuals of ANG sire.

## Discussion

Host factors are fundamental to determine the presence of particular microbial communities in the gastrointestinal tract [14]; however, the interactions of these communities with the host mechanisms responsible for the variations on the metabolic phenotype are completely unknown. Although previous evidence in other species has demonstrated that host factors can contribute in shaping the microbial populations in the GI tract [17], [22], some questions remain unanswered: does sire breed influence bacterial populations? How does this relationship change with diet? And further, how do these interactions relate to variations in basic metabolic processes of the host? Based on our hypothesis that sire breed can impact specific microbial groups present in the rumen of their offspring, as well as their relationships with host ruminal metabolism and phenotypic characteristics, we determined the differences in ruminal bacterial/methanogen populations influenced by sire breed.

Although rumen fluid samples were obtained by oro-gastric tubing since there is the limitation of cannulating a large number of steers to obtain samples from a commercial herd and this method may favour the collection of a larger portion of the bacterial planktonic population in the rumen, there is evidence that rumen samples collected via oral lavage or rumen cannula yield similar results [28]. Hence, the identified phylotypes can represent only the predominant planktonic bacterial and methanogen phylotypes and the sampling method may limit the detection of fibrolytic microborganisms. As in our previous study on the association between bacterial phylotypes and host RFI under different diets [10], similar linkages were observed with host sire breed. A comparison of the most frequent phylotypes present in the offspring of the three sire breeds on each diet showed that under LE diet only ANG progeny had a significantly higher frequency of phylotype occurrence than CHA and HYB progeny, while no exclusive phylotypes for CHA and HYB sire breeds were distinguished on either diet, potentially suggesting that the offspring of these two breeds displayed a larger diversity.

Upon diet switch, cultured and uncultured *Prevotella* sp. were the four most frequent phylotypes in ANG offspring; one phylotype belonging to this genus was also the most frequent in CHA animals. This genus has been reported to be the most abundant bacteria in the rumen [29]. Functional plasticity of this genus has been previously proposed [30]. As a result, and given that it was more frequent in ANG progeny, it is possible that such phylotype plays specialised functions [29], [30] in the ruminal metabolism of this breed. When diet changed, the ANG-associated bacterial phylotypes shifted; hence their adaptive capacity may provide the host with a stable and resilient rumen environment, which in turn will impact the metabolic phenotype. Indeed, high specific activities of particular phylotypes can result in functional significance despite its low numbers [11]. Therefore, the potential functional specialisation of ANG-associated phylotypes might aid in preventing drastic variations on basic metabolic processes, even if significant differences in the frequency of the phylotypes could not be detected. Although *Robinsoniella* sp. was the most frequent phylotype in HYB fed LE, it was also present in HE; similarly, the most frequent phylotype in HE (*Succinivibrio* sp.) was also recorded in 56% of the HYB progeny fed LE diet (Table 2). Thus, HYB offspring did not show any exclusive association with particular bacterial phylotypes under HE diet. In fact, the wide diversity and lack of specialised microbiota in HYB steers on either diet may reflect the absence of cooperative relationships among microbial consortia and even of destabilised population processes [31].

Diet is one of the environmental factors that hinder a complete understanding of the influence of host genetic background in other species, as it is confounding with host factors. To evaluate the effect of the host on the ruminal microbiota, we used independent cohorts of animals of different sire breed, and fed controlled diets. Analysis of the diet-associated variations in ruminal variables and particular microbial phylotypes indeed confirmed that the genetic background of the host might play an important role when differences in those variables are observed. Zhou et al. [25] showed some evidence that diet has impact on the methanogenic community and influences the presence of phylotypes with diverse methanogenic pathways. Variations in the frequency of these methanogen phylotypes might be explained by differences in substrate preferences; the less frequent phylotypes may represent species that adapt to host conditions or particular diets. *Methanobrevibacter olleyae*, *Methanobrevibacter smithii* and *Methanobrevibacter* sp. AbM4 utilise CO_2_-H_2_ and/or formate and acetate for methanogenesis [25]. Phylotypes belonging to *Methanobrevibacter* genus have been found to have a high threshold for H_2_ use [32], [33]. As observed by qPCR, the total population of methanogens was increased in ANG steers under LE diet but remained stable under HE (Tables 6 and 7), suggesting that members of this genus may remain abundant in the rumen and that host characteristics may also influence their prevalence when environmental conditions change [34].

From the bacterial phylotypes selected for validation, *Robinsoniella* sp. was not significantly different among breeds when LE was supplied, but *Succinivibrio* sp. was significantly lower in ANG steers (Table 6). *Succinivibrio* sp. has been observed to have high maintenance coefficients [35] and uses CO_2_ as main substrate. The production of formate by *Succinivibrio* sp. may be a factor in the rate of methanogenesis in the rumen [36], depending on the availability of CO_2_. In ANG progeny methanogens potentially consume more CO_2_ (indicated by the high total methanogen population, Table 4) but methanogenesis might be limited by the high ammonia concentration [33], [37] (Table 5). Taking into account the equal dietary and management conditions, the effect of sire breed on shaping the interactions of the rumen microbiota cannot be neglected.

Interactions within both diets could not be assessed at the same time using Correspondence Analysis, and the data cloud was not conclusive regarding the associations with sire breeds. Thus, the multivariate method outlined allowed us to further pin down additional phylotypes that may be impacted by host factors and ultimately contribute to differences in host performance. Hence, the cluster containing methanogen phylotypes M16 (*Methanobrevibacter gottschalkii*), and M20 (*Methanobrevibacter smithii*) together with bacterial phylotype B62 (*Robinsoniella* sp.) in ANG offspring fed LE diet, is a reflection of the metabolic interactions among microbial consortia. As mentioned above, the interplay among these phylotypes may represent a mutually beneficial commensal relationship, which potentially influences host physiological mechanisms. Methanogenic *Archaea* tolerate higher ammonia by growing within biofilm-like communities [38]. Thus, even the biofilm formation mechanisms might also be different among breeds. Another potential consortium detected was conformed by phylotypes B63 (*Eubacterium* spp.), M17 (*Methanobrevibacter ruminantium*) and M28 (*Methanobrevibacter gottschalkii*), in the three breeds when LE was fed, and in HYB and in ANG progeny on HE diet (Figure 3). Under LE diet, *Eubacterium* sp. proportion tended to be low in ANG, but it was not significantly different among breeds. The presence of these phylotypes clustered together might also represent probable synergic action with other phylotypes; for instance, *Eubacterium* sp. may also be concurrently present with *Succinivibrio* sp. Even if these interactions are observed in the three animal cohorts, the additional cluster of M16-B62-M20 might reflect concomitant associations that impact the host performance and that are influence by host genotype (Table 5, Figure 3). Thus, the complexity of the associations among microbial groups in the rumen might also be influenced by host characteristics.

In summary, our study allowed the exploration of potential relationships among particular microbial phylotypes in cattle differing sire breed and their influence of host metabolic processes and ultimately, in productive performance. The animals sampled in this study are the offspring between Angus, Charolais and Hybrids sires and composite dams, which is not the ideal experimental setting to analyse differences between pure breeds, which has been previously reviewed [9]. Nonetheless, because a large proportion of the genome is shared among the progeny, the use of hybrid individuals is a suitable strategy to observe whether the genetic background had any influence on the presence/absence of microbial phylotypes. This is to say, whether bacterial phylotypes are specific of a breed and whether they may be passed on to the progeny. Additionally, for a robust evidence of the contribution of parental breed to the frequency of bacterial phylotypes in the rumen, research needs to be conducted in a group of purebreds (sires and dams) and their respective progeny. Sample size may bias the underlying associations among phylotypes, as it influences the probability of detecting a higher frequency of phylotype occurrence among breeds. Moreover, several other factors may influence nutrient utilisation and hence the relationships with the phenotypic indicators of feed efficiency [39]. However, similar efficiency has been reported with crossbreds fed high concentrate diet [40]. Whole-genome SNP assessment detected some phylogenetic relationships among European breeds but overall genetic differences between Angus and Charolais individuals [41]; this trend was observed in the clustering of the correlation coefficients, as HYB and CHA shared more similar frequencies of the detected phylotypes in regards to ANG progeny. The identified phylotypes may represent the same species, or even the same strains of different species, and they only account for a small portion of the whole rumen microbiome. We are currently focusing on elucidating the functions of the identified phylotypes associated with sire breeds and high-throughput sequencing techniques will be applied to explore the additional phylotypes present. To our knowledge, this is the first study describing the interactions among microbial profiles, host metabolic phenotype and sire breed. As might be expected from the scope of the work, the above identified correlations among populations may only represent the basis for future research. An improved understanding of the contribution of the rumen microflora to the productive performance of the host will provide insight into the mechanisms that cause the variation between animals in feed utilisation and may ultimately contribute to a decrease in feed costs.

## Materials and Methods

### Animals and Sampling

Animals were selected from a herd of 180 steers raised under feedlot conditions at the Kinsella Research Station, following the guidelines of the Canadian Council on Animal Care [42] and the protocol approved by the Animal Care and Use Committee for Livestock at the University of Alberta. One hundred-eighty steers were used in a study aiming to compare feed efficiency between animals when fed a low energy (LE) vs. a high energy (HE) diet. RFI (residual feed intake) was calculated by linear regression of DMI (dry matter intake) on ADG and MWT (average daily gain and metabolic mid weight), with the residual being the RFI (the difference between the predicted intake and DMI). The grouping was done after calculating RFI, and the high RFI represents the right extreme of the normal curve, while the low RFI represents the left extreme portion of the normal curve. We were mostly interested in the most efficient (highly negative) and most inefficient (highly positive) animals; thus, only the thirty extreme animals on both sides of the curve were taken for further analysis; from these, two were discarded due to saliva contamination in the rumen sample (efficient, L-RFI), two had missing feed efficiency data for the HE diet (efficient, L-RFI) and one (efficient, L-RFI) was removed from the analysis due to low quality of the sample. Although the fingerprinting data was not included in this manuscript, we compared the identified bacterial phylotypes with those identified from solid rumen contents and rumen epithelium. Due to omission in the sampling procedure, six additional individuals (five efficient, L-RFI and one inefficient, H-RFI) were removed from the analysis. In this way, data consisted of forty nine beef steers (10 months old), offspring of a cross between a composite dam line and Angus (ANG, n = 3), Charolais (CHA, n = 3) or University of Alberta hybrid (HYB, n = 24) bulls. The RFI ranking of animals under both LE and HE diets used in this study is listed in supplementary Table S3. The dams used [43] were produced from crosses among three composite cattle lines namely Beef Synthetic 1 (BS1), Beef Synthetic 2 (BS2) and Dairy × Beef Synthetic (DBS). Beef Synthetic 1 was composed of Charolais and 20% Galloway, whereas Beef Synthetic 2 was composed of 60% Hereford with the remaining 40% being other beef breeds. The Dairy × Beef synthetic was composed of 60% dairy breeds (Holstein, Brown Swiss, or Simmental) and 40% beef breeds, mostly ANG and CHA [44]. Average age of dams (∼5 years old) was consistent among each group of steers; therefore, the dam age was not included in the analysis model to adjust for the dam age effect. Thus, the progeny sampled consisted of five steers of ANG sires, nineteen individuals of CHA father and twenty-five animals from HYB sires. The differences in sample size were due to the availability of animals in the extreme low and extreme high groups. Steers grazed with their dams until they were weaned in October of the year before they were slaughtered, and they were averaged 193 d ±12 d of age at the beginning of the trial [45]. Animals were first fed low-energy density (LE) feedlot diet composed of 74% oats, 20% hay, and 6% feedlot supplement [32% CP beef supplement containing Rumensin (400 mg/kg) and 1.5% canola oil (ME 2.6 Mcal/kg)] for 90 days. Following one week of adaptation, animals were switched to a high-energy (HE) density feedlot diet composed of 28.3% oats, 56.7% barley, 10% alfalfa pellets, and 5% feedlot supplement [32% CP beef supplement containing Rumensin (400 mg/kg), and 1.5% canola oil (ME 2.9 Mcal/kg)] for further 90 days. Feed intake data were collected using the GrowSafe automated feeding system (GrowSafe Systems Ltd., Airdrie, AB, Canada).

Rumen fluid samples were collected from all steers via oro-gastric tubing on the same day during the last week of the trial (day 83–90) before feeding using the method described by Hernandez-Sanabria et al. [2].

### Assessment of Ruminal Fermentation Products

Rumen fluid was subjected to VFA analysis using gas chromatography. An enzymatic assay was carried out to measure NH_3_–N (R-Biopharm Roche Inc., South Marshall, MI, USA) as in Hernandez–Sanabria et al. [2]. Proportions of each short-chain VFA relative to the total VFA concentration were obtained and used for the statistical analysis of the microbial metabolites [2], [10]. The rationale behind the use of proportions is to account for the influence of sampling method, dilution by the saliva and differences in fermentation stage due to the time elapsed since last meal. Differences in VFA composition and NH_3_–N between breeds (ANG, CHA or HYB) were detected using a mixed model in SAS [46]. Significance was assumed at the *P*<0.05.

### DNA Extraction and Phylotype Analysis

Total DNA extraction was performed as per Guan et al. [9] and Hernandez-Sanabria et al. [10]. The concentration and quality of DNA were measured based on the absorbance at 260 and 280 nm in a Nanodrop ND 1000 spectrophotometer (NanoDrop Technologies, Wilmington, DE, USA). Fifty ng of total DNA were used as template for PCR amplifications of the V2–V3 region of the bacterial 16S rRNA gene (∼200 bp) using universal bacterial primers HDA1-GC/HDA-2 [47]. Primers targeting the V3 region of the 16S rRNA gene were designed for ruminal methanogenic community profiling [25]. Conditions for the amplification, purification of PCR products, as well as for the cloning and sequencing of all bacterial and methanogen PCR–DGGE bands have been reported [2], [25]. Similarities among all bacterial and methanogenic phylotypes from the three breeds (ANG, CHA or HYB) and within each diet were calculated using the Dice similarity coefficient (*D_sc_*) in BioNumerics software v6.5 (Applied Maths, Austin, TX, USA). Hierarchical cluster comparisons of the similarity matrices were generated using the unweighted pair–grouping method (UPGMA) at 1% position tolerance, to generate a binary matrix of band classes. Multi–dimensional Scaling (MDS) tool was used to spatially display bacterial and methanogen profiles and to observe clustering trends between breeds under each diet.

Bacterial and methanogen phylotypes from all profiles on each breed were obtained with BioNumerics Software. Following the procedure described by Hernandez-Sanabria et al. [2], new categories including all the detected phylotypes on both diets were created for the three breeds. Associations between phylotypes and sire breed were identified using a Chi-square analysis (PROC CATMOD in SAS). Frequency of sire breed–associated phylotypes within diet was compared using 3×2 contingency tables of cross classifications containing the frequencies of the phylotypes on each diet, obtained with the FREQ procedure in SAS. Diet–associated phylotypes were obtained for each breed, upon creation of 3×2 contingency tables. Table probabilities were calculated using Fisher Exact test when the count of any of the cells was below 5, otherwise Chi-square was preferred. For each phylotype, pairwise comparisons between breeds (ANG vs. CHAR, ANG vs. HYB and CHAR vs. HYB) were performed using the FREQ procedure in SAS, to detect significant differences in phylotype frequency between any pair of breeds. Significant differences were declared at *P*<0.05.

### Quantitative Real-time PCR

For the quantification of the total bacterial 16S rRNA gene copy number, a standard curve was constructed using serial dilutions of plasmid DNA of a *Butyrivibrio hungateii* clone [4], [10]. Standard curves for quantification of total methanogen population were constructed using a serial dilution of plasmid DNA from a clone identified as *Methanobrevibacter* sp. AbM4 [25]. The mass concentration of the PCR products was measured by spectrophotometry and converted to the molecule concentration using the equation: DNA (number of molecules) = (NL×A×10^−9^)/(660×n), where NL is the Avogadro constant (6.02×10^23^ molecules per mol), A is the molecular weight of the molecule in the standard, and n is the length of the amplicon (bp) [4]. The copy numbers of the targeted bacterial and methanogen 16S rRNA genes per ml of rumen fluid were calculated using the equation: (QM×C×DV)/(S×V), where QM is the quantitative mean of the copy number, C is the DNA concentration of each sample, DV is the volume of the total extracted DNA, S is the DNA amount (ng) subjected to DGGE analysis and V is the initial volume of rumen fluid subjected to DNA extraction, multiplied by the dilution factor [10], [25].

To validate the relationship between specific bacterial phylotypes and sire breed, within each diet, total rumen fluid DNA from all 49 steers was subjected to qPCR analysis to estimate the copy number of the 16S rRNA gene for each of the following bacterial phylotypes: *Robinsoniella peoriensis* (associated with the three breeds in LE), *Eubacterium sp.* (associated with ANG steers in both diets) and *Succinivibrio dextrinosolvens* (associated with HYB steers in HE). Primer Express v2.0 (Applied Biosystems, Foster City, CA, USA) was used to design primers targeting the sequence of the phylotypes related to the genus *Robinsoniella*, as outlined by Hernandez-Sanabria et al. [10]. The qPCR assays for *Eubacterium rectale* [48] and *Succinivibrio dextrinosolvens* [29] have been previously described. Similarity at 97% was used as cut-off for species level and 93% similarity as cut-off for genus level [49]. The sequences obtained in this study had 95% identity with *Eubacterium rectale* and *Succinivibrio dextrinosolvens*; thus, *Eubacterium* sp. and *Succinivibrio* sp. were used to represent the corresponding phylotypes. The proportion of each phylotype was obtained as described by Hernandez-Sanabria et al. [10]; efficiencies of qPCR were calculated from the given slopes in StepOneplus software, using the following equation: E = [10(^−1/slope^) –1]×100%. qPCR was performed using a StepOnePlus real-time PCR system and SYBR GREEN chemistry (Applied Biosystems, Foster City, CA, USA). Data generated from reactions with efficiencies between 90 and 110% were used for further analysis [10].

Analysis of variance using a mixed model in SAS was used to identify differences in total bacterial 16S rRNA gene copy number and in the proportion of each of the three specific bacterial phylotypes (*Succinivibrio* sp., *Eubacterium* sp., and *Robinsoniella* sp.) between breeds (ANG, CHAR and HYB), within each diet (LE/HE). Correlations among proportions of each bacterial phylotype, total copy number of bacteria and methanogens, ruminal metabolites and phenotypic traits (Residual Feed intake, RFI; Average Daily Gain, ADG; Dry Matter Intake, DMI; and feed Conversion Ratio, FCR), were determined using the CORR procedure in SAS, within breed for both diets. In addition, correlations among the above measurements were performed within diet, for the three breeds separately. Significance was assumed when *P*<0.05.

### Statistical Analysis

Bacterial communities in the rumen can be compared among samples using the multivariate statistics approaches that community ecologists have employed to study macro-organisms [50] and microbiota from diverse environments [51]. Principal Component Analysis (PCA) was used to initially assess the potential relationships among bacterial and methanogen phylotypes present on each breed, as well as to observe how diet may impact such interplay. A matrix containing the frequencies of each bacterial and methanogen phylotypes (obtained from the contingency tables) or zero, to indicate that the band was not observed in a particular breed, was created for each diet and analysed using PRINCOMP in SAS. As PCA might force linear relationships and mask ecologically important relationships among particular phylotypes, it was considered only a method to overview the potential influence of breed, within a particular diet, on the whole microbial ecosystem.

An additional ordination procedure, Correspondence Analysis (CA) was employed to visualise broad relationships among specific bacterial/methanogen phylotypes. Correspondence analysis is a statistical tool for the graphical display of contingency tables [52]; it produces two-dimensional plots of the data variation, which allows observing overlaps between variables [23]. Structural relationships among frequencies were displayed in a data cloud, where Dimension 1 indicated the frequency of either methanogen or bacterial phylotypes (column coordinates) while Dimension 2 depicts the associations of these observed frequencies and breed (row coordinates). The summary of these relationships was shown, and phylotypes were disseminated in the plot, in close relation with the breed where they were more frequent. As the CA did not satisfactorily verify our hypothesis related to the breed influence on the microbial interplay in the rumen, further methodology was explored.

Frequency data from the contingency tables was analysed using a two-way hierarchical cluster analysis, and displayed as a heat map of Spearman correlation coefficients [24]. To test whether the frequency of the 113 microbial phylotypes detected was significantly different on each breed/diet, pairwise correlations exceeding 0.5 were recorded. Values were represented by colour intensities, according to the Z scores, where Z = (observed value – mean)/standard deviation. Lighter intensities indicate that frequencies of a particular phylotypes are higher than the average frequency of all the phylotypes [53]. Dark spots indicate self-correlations [24].

## Supporting Information

Table S1 Correlation (r) of fermentation metabolites in the rumen of ANG,CHA, and HYB steers under LE diet with phenotypic indicators of metabolic differences (RFI, DMI and FCR) and bacterial and methanogen population (n = 5), ***p<0.0001, **p<0.05, *trend.
(DOC)

Table S2 Correlation (r) of fermentation metabolites in the rumen of ANG,CHA, and HYB steers under HE diet with phenotypic indicators of metabolic differences (RFI, DMI and FCR) and bacterial and methanogen population (n = 5), ***p<0.0001, **p<0.05, *trend.
(DOC)

Table S3 **Animals included in our study, the breed of the parent sire and the RFI classification under low energy (LE) and high energy (HE) diets.**
(DOC)
