# Supplementary material for: Influence of Sire Breed on the Interplay among Rumen Microbial Populations Inhabiting the Rumen Liquid of the Progeny in Beef Cattle
Source: PLoS One. 2013 Mar 8;8(3):e58461. doi: 10.1371/journal.pone.0058461 (PMC3592819; doi:10.1371/journal.pone.0058461)
Supplement: Table S1 — Correlation (r) of fermentation metabolites in the rumen of ANG,CHA, and HYB steers under LE diet with phenotypic indicators of metabolic differences (RFI, DMI and FCR) and bacterial and methanogen population (n = 5), ***p<0.0001, **p<0.05, *trend. (DOC) [file pone.0058461.s001.doc]

Table S1. Correlation (r) of fermentation metabolites in the rumen of ANG,CHA, and HYB steers under LE diet with phenotypic indicators of metabolic differences (RFI, DMI and FCR) and bacterial and methanogen population (n = 5), ***p<0.0001, **p<0.05, *trend.

| **Variable** | **Total bacteria** | ***Succinivibrio* sp.** | ***Eubacterium* sp.** | ***Robinsoniella* sp.** | **Total Methanogens** |
| --- | --- | --- | --- | --- | --- |
| **Butyrate** | -0.394* (CHA)  0.544** (HYB) | NS | NS | NS | -0.480** (CHA) |
| **Valerate** | NS | NS | NS | NS | -0.369* (HYB) |
| **Isovalerate** | NS | NS | NS | 0.423** (HYB) | NS |
| **Total VFA** | NS | 0.877** (ANG) | NS | NS | NS |
| **Branched VFA : Straight VFA ratio** | NS | 0.919** (ANG) | NS | NS | NS |
| **NH_3_-N** | NS | 0.510** (CHA) | 0.488** (HYB) | 0.903**(ANG)  0.394* (HYB) | -0.381* (HYB) |
| **RFI** | NS | NS | -0.820*(ANG) | NS | 0.365* (HYB) |
| **DMI** | NS | NS | -0.974**(ANG) | NS | NS |
| **FCR** | NS | NS | NS | NS | -0.816* (ANG) |
