# Supplementary material for: Influence of Sire Breed on the Interplay among Rumen Microbial Populations Inhabiting the Rumen Liquid of the Progeny in Beef Cattle
Source: PLoS One. 2013 Mar 8;8(3):e58461. doi: 10.1371/journal.pone.0058461 (PMC3592819; doi:10.1371/journal.pone.0058461)
Supplement: Table S3 — Animals included in our study, the breed of the parent sire and the RFI classification under low energy (LE) and high energy (HE) diets. (DOC) [file pone.0058461.s003.doc]

Table S3. Animals included in our study, the breed of the parent sire and the RFI classification under low energy (LE) and high energy (HE) diets.

| **Animal ID** | **Breed of sire** | **RFI category**  **(LE diet)** | **RFI category**  **(HE diet)** |
| --- | --- | --- | --- |
| 1 | CHA | H | M |
| 11 | HYB | H | H |
| 13 | HYB | L | M |
| 15 | CHA | M | M |
| 23 | HYB | L | L |
| 33 | ANG | L | L |
| 45 | HYB | M | L |
| 63 | CHA | M | L |
| 65 | HYB | L | M |
| 69 | CHA | L | H |
| 71 | ANG | M | H |
| 75 | CHA | L | L |
| 91 | HYB | L | L |
| 93 | HYB | M | H |
| 95 | HYB | H | H |
| 97 | HYB | H | M |
| 101 | CHA | L | L |
| 111 | CHA | L | L |
| 119 | ANG | L | L |
| 121 | CHA | L | M |
| 129 | HYB | L | L |
| 151 | CHA | H | M |
| 153 | CHA | M | H |
| 159 | HYB | H | M |
| 161 | HYB | H | M |
| 163 | HYB | H | M |
| 167 | HYB | H | H |
| 173 | HYB | H | H |
| 183 | CHA | M | H |
| 205 | ANG | L | L |
| 209 | CHA | M | L |
| 225 | CHA | H | H |
| 249 | HYB | L | M |
| 251 | HYB | M | H |
| 271 | HYB | L | L |
| 279 | HYB | H | L |
| 287 | ANG | H | H |
| 293 | HYB | L | M |
| 311 | HYB | H | M |
| 401 | HYB | M | M |
| 403 | HYB | H | M |
| 407 | HYB | H | M |
| 411 | CHA | H | M |
| 423 | CHA | L | M |
| 427 | CHA | H | M |
| 437 | CHA | M | M |
| 439 | CHA | H | M |
| 451 | CHA | L | L |
| 465 | HYB | M | H |
